# A Comparison of the Electrochemical Oxidative Dissolution of Pyrite and Chalcopyrite in Dilute Nitric Acid Solution

**DOI:** 10.1002/open.202400053

**Published:** 2025-04-02

**Authors:** Samaneh Teimouri, Johannes Herman Potgieter, Caren Billing

**Affiliations:** ^1^ Sustainable and Innovative Minerals and Metals Extraction Technology (SIMMET) Group School of Chemical and Metallurgical Engineering University of the Witwatersrand Private Bag X3 Wits 2050 South Africa; ^2^ Molecular Sciences Institute School of Chemistry University of the Witwatersrand Private Bag X3 Wits 2050 South Africa

**Keywords:** Pyrite, Chalcopyrite, Oxidative dissolution, Nitric acid, Cyclic voltammetry (CV), Electrochemical impedance spectroscopy (EIS)

## Abstract

Understanding the oxidation of sulfidic minerals, especially those of pyrite and chalcopyrite, under acidic conditions has important outcomes, such as exposing any encapsulated gold not recovered by traditional cyanidation processes. This study focused on the electrochemical oxidation of pyrite and chalcopyrite in a 0.5 M nitric acid solution. Electrochemical techniques were employed, using the minerals as working electrodes. Cyclic voltammetry (CV) was performed to detect redox processes and resulting products were suggested. Electrochemical impedance spectroscopy (EIS) was run at specific potentials corresponding to oxidation processes detected to further probe the reaction mechanism. For pyrite at low anodic potentials (0.4–0.6 V vs Ag/AgCl), Fe_1‐x_S_2_ and Fe(OH)_3_ with a sulfur‐rich layer which forms S^0^ accumulates on the electrode surface, leading to diffusion controlled dissolution processes. Above 0.7 V, the pyrite is fully oxidised, eradicating the diffusion barrier and extensive oxidation occurs at high potentials (0.9 V). Similar processes occurred for chalcopyrite with mainly iron‐deficient sulfides (like Cu_1‐x_Fe_1‐y_S_2‐z_, CuS_2_, CuS) forming at low potentials (0.3–0.5 V), and S^0^ partially covering the surface causing a diffusion barrier. Increasing the potential to beyond 0.7 V leads to these layers converting to soluble species.

## Graphical abstract

This research was primarily driven by the aim to extract residual encapsulated gold in sulfidic minerals, which would require the dissolution of the mineral. The electrochemical oxidation of pyrite and chalcopyrite in 0.5 M nitric acid was investigated here, using cyclic voltammetry and electrochemical impedance spectroscopy, to see if there are any differences compared to when sulfuric acid is used.

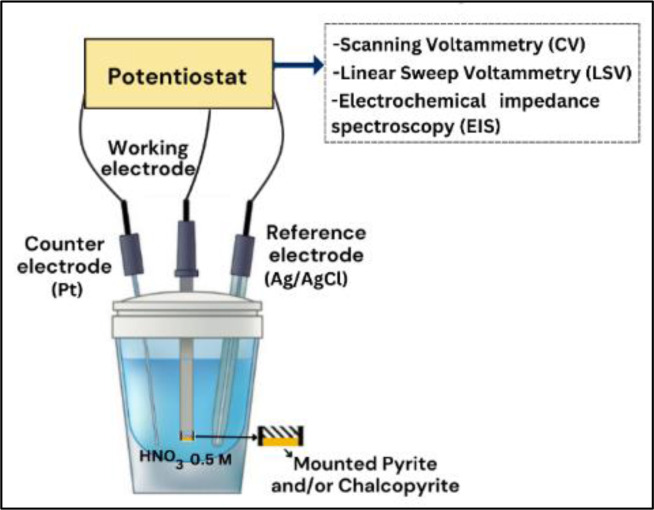

## 1 Introduction

Pyrite and chalcopyrite, which often occur together, are widespread sulfide minerals present in the earth's crust.[^1^, ^2^] Pyrite (FeS_2_), the most common iron sulfide mineral, is used in the production of sulfur and sulfuric acid.^[3]^ Chalcopyrite (CuFeS_2_), the most abundant copper mineral, forms the basis of copper production. Researchers recently developed novel applications of pyrite and chalcopyrite, based on their non‐toxic, semi‐conductive properties, their abundance and low material cost.^[4]^ These minerals have various electrochemical applications, such as in solar energy collectors in solar panels, as solid‐state sensor materials,^[5]^ as a depolarizer anode in hydrogen production,^[6]^ and as a cathode or an anode material for lithium‐based batteries.^[7]^

Pyrite and chalcopyrite often processed to extract the associated precious metals, particularly gold. The oxidation of these sulfidic minerals is important for enhancing gold extraction from refractory sulfidic ores and mine tailings, since pyrite and chalcopyrite oxidation expose encapsulated gold.[^8^, ^9^, ^10^] On the other hand, there are some environmental problems caused by sulfide minerals. In mining operations and tailings, exposure of sulfide minerals to air and water generates acid mine drainage (AMD) due to oxidative dissolution. This can lead to groundwater contamination.[^11^, ^12^] The co‐existence of pyrite in coal also leads to the emission of sulfur dioxide at power stations in which coal is burned for power generation.[^13^, ^14^]

Global industrialization, automation, and the depletion of high‐grade ores have increased demand for valuable metals, often outpacing supply.^[15]^ To address this, focus has shifted to extracting metals from sulfidic refractory low‐grade ores, mine tailings, and industrial waste. This has prompted investigations into the electrochemistry of pyrite and chalcopyrite, using cyclic voltammetry (CV) to identify redox processes at specific potentials.[^16^, ^17^]

Despite extensive research, the redox reactions, product compositions, passive surface layers, and intermediates in the oxidative dissolution of these minerals remain debated. Earlier studies postulated that the passive layer and the intermediates resulting from the oxidative dissolution of pyrite and chalcopyrite may consist of metal‐deficient sulfides such as pyrrhotite (Fe_1‐x_S_2_), copper‐rich species (Cu_1‐x_Fe_1‐y_S_2‐z_, Cu_2_S, CuS), and sulfur segments (elemental sulfur (S^0^) and polysulfides S_n_^2−^, n>2), as well as iron‐rich layers composed of Fe_2_O_3_, FeO, Fe(OH)_3_, and Fe(OH)_2_.[^17^, ^18^, ^19^, ^20^]

Tu et al.^[18]^ studied pyrite oxidation at pH=2 (adjusted with 10 % H_2_SO_4_) finding that at 0.5 V vs saturated calomel reference electrode (SCE), Fe(OH)_3_ and elemental sulfur (S^0^) formed, covering the surface of the pyrite electrode, which resulted in reactions being diffusion‐limited. At 0.6 V (vs SCE), the sulfur‐rich layer converted to crystalline S_8_, allowing continued oxidation. At 0.8 V (vs SCE), Fe(OH)_3_ and SO_4_^2−^ formed, along with polysulfides (S_n_^2−^) and an iron‐rich layer consisting of FeO and Fe_2_O_3_ which accumulated on the surface of the pyrite.^[18]^ Zhang et al.^[21]^ examined the dissolution of pyrite, pyrrhotite, chalcopyrite, bornite, and chalcocite in acidic sulfate media using CV, noting that an inhibitory layer forms during initial oxidation, which can be removed with higher oxidation potentials, facilitating further dissolution.^[21]^

Lazaro & Nicol^[22]^ studied the anodic dissolution of chalcopyrite in 0.5 M sulfuric acid solution. They found evidence of the formation of Cu^2+^, Fe^2+^, and soluble sulfur species (H_2_S) at the early stage of oxidative dissolution of chalcopyrite at 60 °C. Ghahremaninezhad et al.^[19]^ employed potentiodynamic polarization, electrochemical impedance spectroscopy (EIS), and Mott‐Schottky methods to analyze chalcopyrite oxidation, finding a thin Cu_1‐x_ Fe_1‐y_ S_2_ (y>x) layer that thickened from OCP ~−0.235 V to 0.10 V vs MSE (saturated Hg/Hg_2_SO_4_ electrode; ~0.20 V to 0.53 V vs Ag/AgCl). At 0.30 V vs MSE (~0.73 V vs Ag/AgCl), this layer partially dissolved, leading to Cu_1‐x‐z_S_2_ formation. At higher potentials (0.30–0.42 V), both these layers dissolved, and extensive dissolution of the chalcopyrite electrode begins. Further increasing the potential to 0.75 V vs MSE (~1.18 V Ag/AgCl) led to the formation of CuS and Fe_2_(SO_4_)_3_ concomitantly.^[19]^

Old mine dumps in South Africa still contain residual gold, which is recovered by DRDGold at their ERGO plant in Johannesburg. Much of this gold is refractory, primarily encapsulated in pyrite and, to a lesser extent, chalcopyrite. Teimouri et al.^[23]^ examined the dissolution kinetics of these mine tailings, predominantly pyrite, in nitric acid as a potential method for enhancing gold recovery. Most previous studies focused on sulfuric acid solutions, and it remains uncertain whether the dissolution mechanisms would be similar in nitric acid.

This research aims to independently confirm the electrochemical behaviour of pyrite and chalcopyrite in 0.5 M nitric acid. Cyclic voltammetry (CV) scans were conducted at various scan rates and potential ranges to identify possible redox reactions. Linear sweep voltammetry (LSV) focused on oxidative dissolution potentials, guiding the selection of potentials for further measurements using electrochemical impedance spectroscopy (EIS). This approach seeks to provide new insights into recovering gold residues from mine tailings and determine if dissolution mechanisms differ between nitric and sulfuric acids. Additionally, it aims to clarify the differences in dissolution behaviour and mechanisms of pyrite and chalcopyrite, ultimately linking these findings to the kinetics of their dissolution.

Additionally, the dissolution behaviour of pyrite and chalcopyrite is of particular interest for biomining applications, where bacteria are often used for leaching metals from sulfidic ores.[^24^, ^25^, ^26^] In such cases, both the mineralogical composition of the material and the governing mechanism influence the dissolution kinetics. In particular, the kinetics and mechanism of copper dissolution plays an essential role, as copper can be toxic to bacteria above limiting concentrations. This work therefore should be highly relevant for both bioleaching operations involving pyrite and chalcopyrite.

## Experimental

### Preparation and Characterization of Pyrite and Chalcopyrite Electrodes

To create the working electrode from the minerals (pyrite and chalcopyrite), a small piece of each mineral was encapsulated in an epoxy resin matrix. One end of the mineral was connected to a copper wire using silver glue, while the surface on the other end was left exposed. After the epoxy resin dried overnight, the exposed surface area of the pyrite/chalcopyrite was polished with a rotating disc covered with consecutively finer‐grained silicon carbide papers. To smooth the surface and remove any surface defects, the exposed surface was polished using a rotating disc covered with a soft cloth impregnated with alumina (0.5 μm) mixed with deionized water. After polishing, the electrodes were thoroughly rinsed with deionized water to remove any alumina particles. The exposed surface area of both pyrite and chalcopyrite electrodes was measured under a microscope connected to Nicon EIS‐Elements software. The surface area for pyrite and chalcopyrite were 53.4 mm^2^ and 56.7 mm^2^, respectively. The preliminary phase analysis for pyrite and chalcopyrite used to make the electrodes was analysed with X‐ray diffraction (XRD) (Bruker D2 Phaser). In addition, scanning electron microscopy and energy dispersive spectrometry (SEM‐EDS) (ZEISS Sigma) to determine their chemical composition was also performed.

### Electrochemical Measurements

The electrochemical techniques applied in this work include cyclic voltammetry (CV) to gain an oversight of the redox processes and their kinetics, linear sweep voltammetry (LSV) to obtain better estimates of applied potentials to be used in the electrochemical impedance spectroscopy (EIS) measurements, and EIS to further probe the reaction mechanism. To ensure the initial stability of the pyrite and chalcopyrite electrodes, they were immersed in 0.5 M HNO_3_ solution as the electrolyte, and the open circuit potential (OCP) was measured. This was done for 120 min (until a stable value was maintained) before the EIS measurements were initiated at each DC bias potential for each of the electrodes. The cyclic voltammograms (CV) of pyrite and chalcopyrite in 0.5 M HNO_3_ solution were obtained using a Metrohm Autolab PGStat10 potentiostat controlled by General Purpose Electrochemical (GPE) software connected to a typical three‐electrode electrochemical cell. The prepared pyrite or chalcopyrite working electrode (WE) was used along with a Pt counter electrode and Ag/AgCl (3 M KCl) reference electrode (both supplied by Metrohm). The WE was polished before each set of experiments. All potential values quoted in this work are with respect to this reference electrode and a potential step of 4 mV was used for all voltammetric measurements. The LSV was measured in the potential range from −0.1–0.9 V (vs Ag/AgCl). The EIS measurements were conducted using a BioLogic VMP300 potentiostat‐frequency response analyser (FRA) under DC bias at different potentials in the frequency range of 10^−2^–10^6^ Hz with 10 mV amplitude. The EC‐Lab Z‐fit software was used to fit the obtained Nyquist plots. All experiments were conducted at room temperature in a static solution.

## 2 Results and Discussion

### 2.1 Characterization of Pyrite and Chalcopyrite Electrode

**Figure 1:**
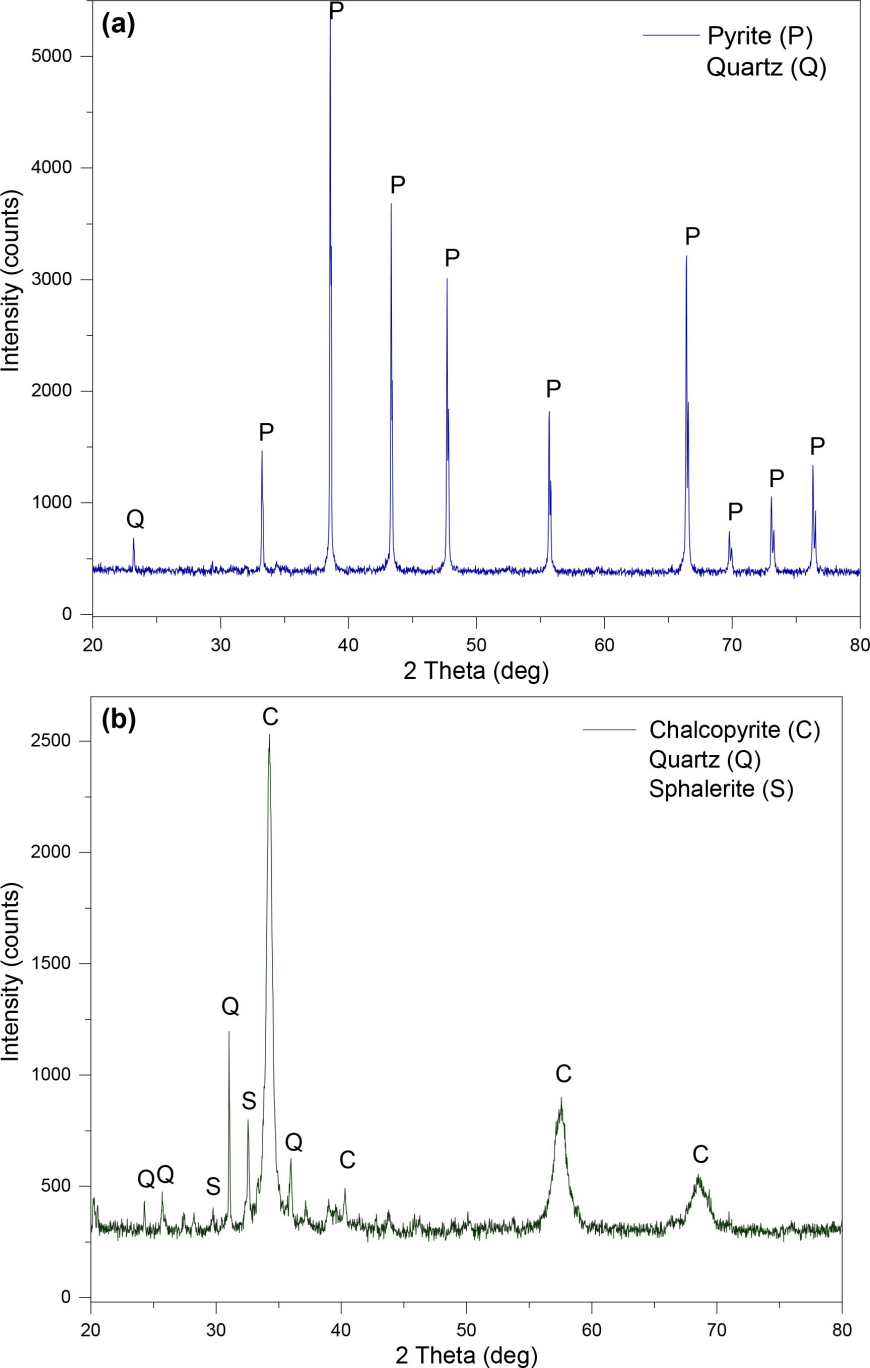
XRD patterns of (a) pyrite and (b) chalcopyrite minerals.

**Figure 2:**
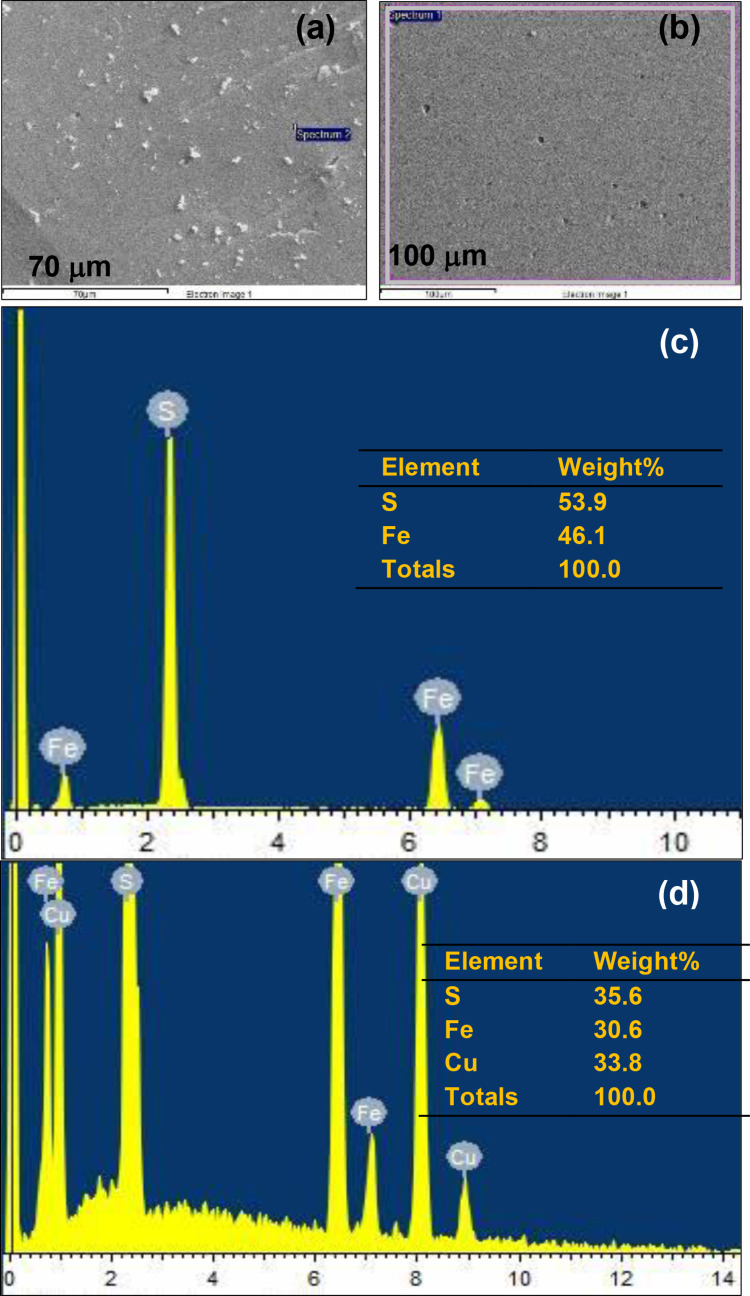
SEM‐EDS of (a,c) pyrite and (b,d) chalcopyrite electrode.

The pyrite and chalcopyrite minerals that were used to make the electrodes, were analysed by XRD, as well as SEM‐EDS to identify the main phases, the chemical composition and purity of the minerals.^[27]^ Figure 1(a‐ pyrite and b‐ chalcopyrite) represents the XRD patterns showing mainly the presence of pyrite and chalcopyrite, with some impurities such as quartz (SiO_2_) in both minerals and sphalerite (zinc sulfide mineral with (Zn, Fe)S chemical composition) in the chalcopyrite. Figure 2(a and c) shows the SEM‐EDS of pyrite (FeS_2_) (formula weight=119.98 g) with the detection of 53.9 % sulfur (S) and 46.1 % iron (Fe) (as wt %), which is close to the theoretical percentage of the main elements for pyrite, with sulfur 53.5 % and iron 46.6 %.^[28]^ Figure 2(b and d) shows the SEM‐EDS analysis of chalcopyrite (CuFeS_2_) (formula weight=183.5 g), and identified 35.6 % sulfur (S), 30.6 % iron (Fe), and 33.8 % copper (Cu) (as wt %), which is again close to the theoretical percentages of these main elements with sulfur, iron, and copper as 34.9 %, 30.4 %, and 34.6 % (wt %),[^29^, ^30^] respectively.

### 2.2 Cyclic Voltammetry (CV) for Pyrite

**Figure 3:**
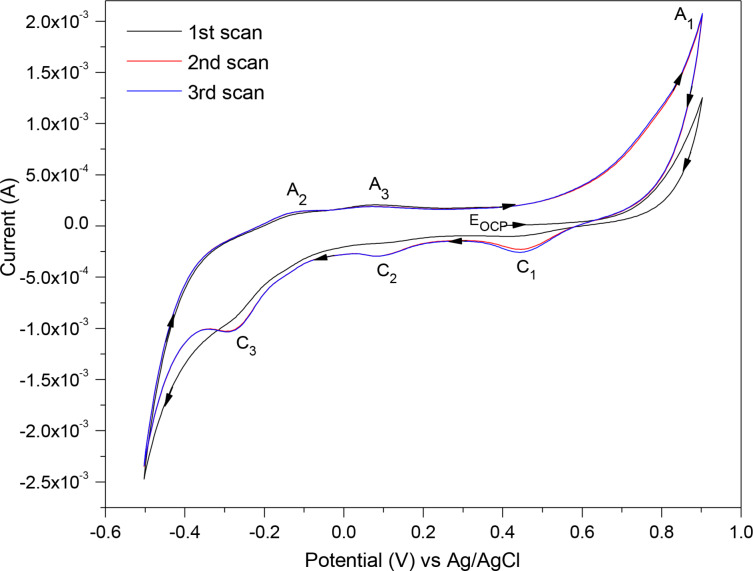
A three‐cycle CV scan of pyrite mineral as the WE with 1.5 V/s scan rate in 0.5 M HNO_3_. The arrows on the scan show the direction of the applied potential.

A typical three‐cycle CV scan of a freshly polished pyrite electrode as the WE in 0.5 M HNO_3_ is presented in Figure 3, which indicates that after the initial scan, the pyrite electrode is stable and yields reproducible scans. The CV scan was started at E_OCP_ (~0.4 V) towards an anodic direction at a scan rate of 1.5 V/s and data were collected within the potential range of +0.9 to −0.5 V. In the anodic (oxidation) section, three anodic peaks were detected, labelled A_1_, A_2_, and A_3_, and correspondingly, in the cathodic (reduction) part, three cathodic peaks C_1_, C_2_ and C_3_ appeared.

**Figure 4:**
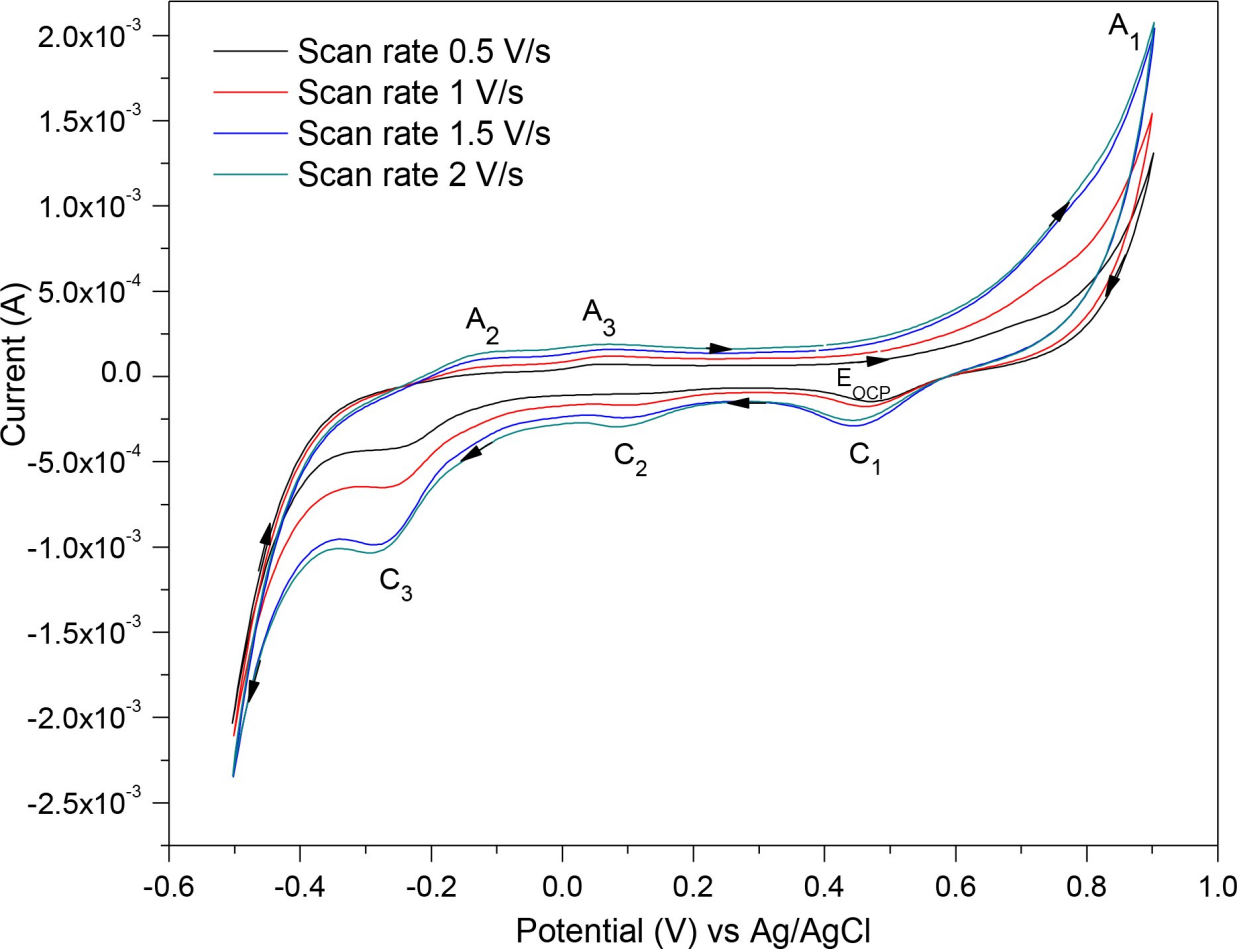
The CV scans for pyrite as the WE at different scan rates in 0.5 M HNO_3_.

Figure 4 represents the voltammograms (for the third cycle) of pyrite each at a different scan rate of 0.5, 1, 1.5 and 2 V/s. As illustrated, increasing the scan rate from 0.5–2 V/s, increased the current response of the redox peaks. The peaks were not clearly distinguishable at lower scan rates (such as 0.05 V/s); even at a scan rate of 0.5 V/s (Figure 4) the oxidation peaks A_2_ and A_3_ are subtle. Fast scan rates allow for the detection of transient species at higher concentrations before they undergo chemical reaction or diffusion which decreases their concentrations at the electrode surface. Hence the scan rate of 2 V/s which led to pronounced redox peaks was applied in the subsequent CV scans.

#### 2.2.1 Effect of Changing the Potential Range on Pyrite

**Figure 5:**
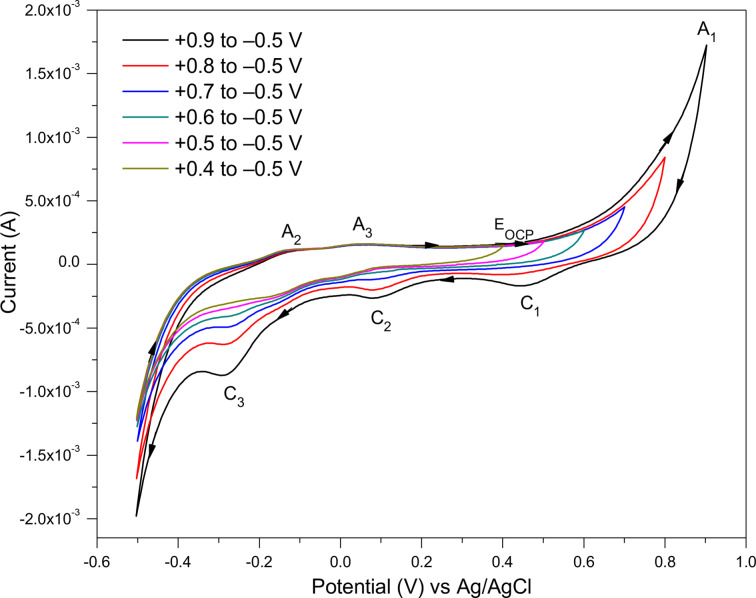
Changing the potential range on the positive side of the pyrite CV scan (the 3^rd^ cycle) measured at 2 V/s scan rate in 0.5 M HNO_3_.

The effect of changing the potential range on the positive side from 0.9, 0.8, 0.7, 0.6, 0.5 and 0.4 V while keeping the cathodic potential fixed at −0.5 V on the CV scans of the pyrite electrode, is illustrated in Figure 5. Altering the positive potential to a smaller range (+0.8 V) first affects the anodic peak A_1_ and the cathodic peak C_1_. By further shortening the positive potential range to +0.6 V the cathodic peak C_2_ becomes very small, and then the peak C_3_ by reducing the range to +0.5 V. Therefore, when the potential range becomes shorter the associated redox reactions cannot be driven fully, hence the related redox peaks appear smaller. This illustrates a clear association between the redox process at A_1_ and C_1_, as well as at C_2_, and C_3_ with A_2_, and A_3_.

**Figure 6:**
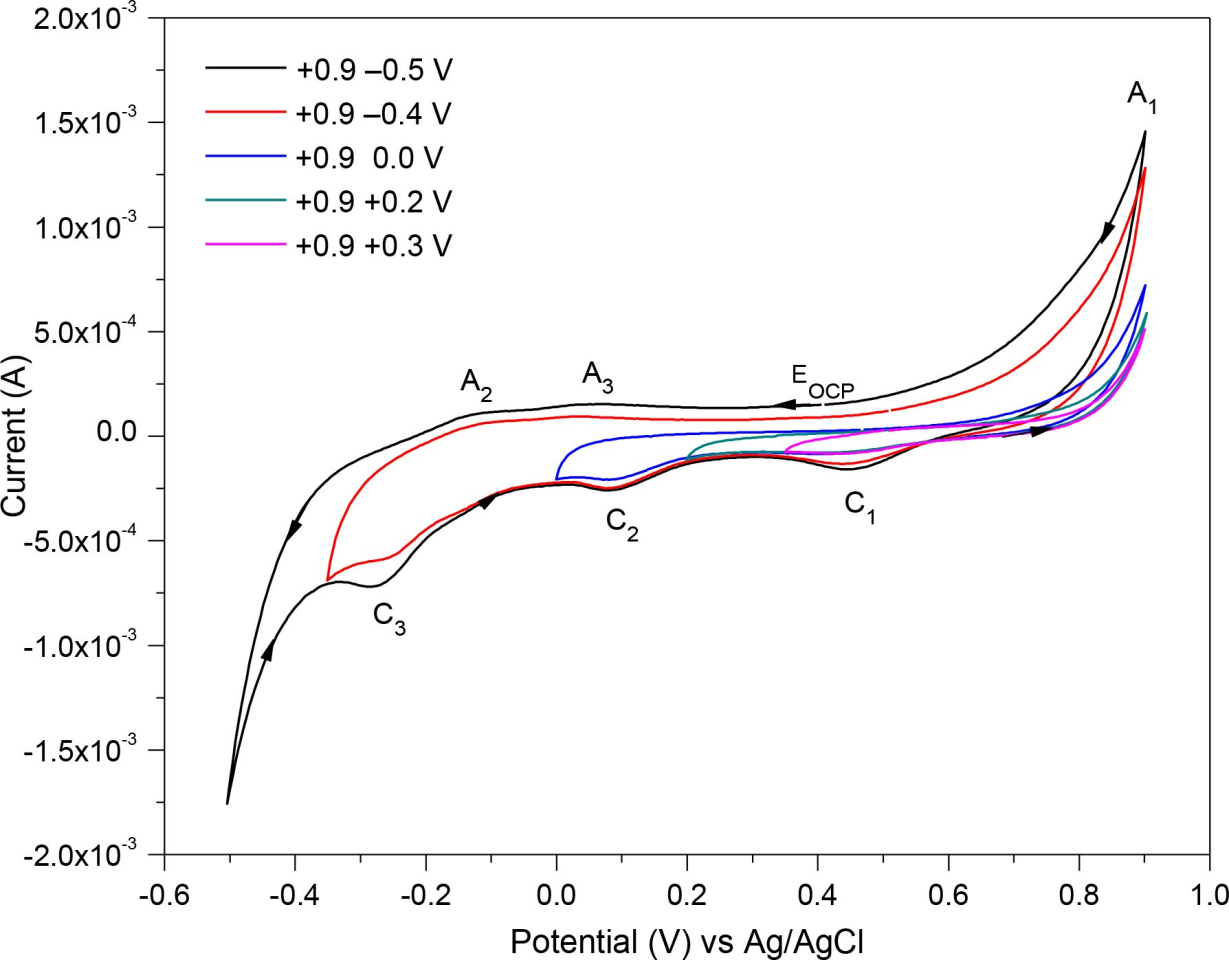
Changing the potential range on the negative side of the pyrite CV scan (the 3^rd^ cycle) measured at 2 V/s scan rate in 0.5 M HNO_3_.

To determine the effect of sweeping to different negative potentials, the direction of the scan was reversed by starting at E_OCP_ and moving to −0.5, −0.4, 0.0, +0.2 and +0.3 V, and then sweeping to a fixed positive potential of +0.9 V, as shown in Figure 6. Making the negative potential smaller (to −0.4 V) the cathodic peak C_3_ and the related anodic peaks A_2_ and A_3_ become smaller. By further reducing the range to 0.0 V, the cathodic peaks C_2_ and C_1_ become smaller, and the anodic peak A_1_ is also affected. With the cathodic vertex reduced to +0.2 V, no reduction peaks appeared and the A_1_ peak became even smaller. Thus, at a shorter negative potential range, the cathodic reactions do not occur completely, consequently the related cathodic peaks (C_3_, C_2_ and C_1_) and the correlated anodic peaks are smaller.

#### 2.2.2 Interpretation of the Redox Peaks in Pyrite CV Scan

The current increased exponentially during the anodic sweep from E_OCP_=~+0.4 V to +0.9 V (see Figure 3). At the highest potential (+0.9 V) at peak A_1_, a complete oxidation of FeS_2_ to Fe(OH)_3_/Fe^3+^ and SO_4_^2−^ with several electrons released is expressed in reaction 1. This can be observed by the sharp escalation of current for a small increment of the potential in the voltammogram.[^18^, ^21^] No oxygen evolution was observed at +0.9 V which would cause bubble formation on the electrode surface (this was the case even when the electrode was maintained at this potential for longer periods while doing EIS measurements). For successive scans, at this high potential (+0.9 V), the previously formed iron deficient sulfide (Fe_1‐x_S_2_) and S^0^ (formed during partial oxidation of pyrite at 0.05 V at A_3_) can further oxidise based on reactions 2 and 3.[^30^, ^31^] Visual scrutiny after the CV scan showed some dark purple/black substance(s) covering the surface of the pyrite electrode. This is likely evidence of formed ferric hydroxide Fe(OH)_3_ as confirmed in research by Tu et al. 2017.^[18]^

(1)

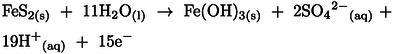



(2)

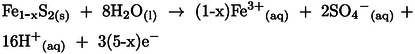



(3)

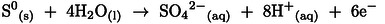




Reversal of the potential in the cathodic direction results in three reduction peaks. The first reduction peak C_1_ at +0.45 V is assigned to the reduction of Fe(OH)_3_/Fe^3+^ to Fe^2+^, i. e. a one‐electron reduction based on reactions 4 and 5.[^18^, ^21^, ^32^, ^33^]
(4)

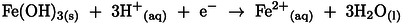



(5)

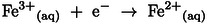




The second reduction peak C_2_ detected at ~+0.1 V represents the reduction of FeS_2_ to FeS and H_2_S based on reaction 6[^18^, ^21^]: 
(6)

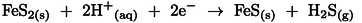




The third cathodic peak C_3_ detected at ~−0.25 V represents the reduction of S^0^ (formed during partial oxidation of pyrite at A_3_) to H_2_S as given by reaction 7.[^18^, ^21^, ^34^] The current produced at the negative vertex potential (−0.5 V) is associated with hydrogen evolution. 
(7)

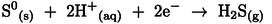




When the scan was reversed to the positive potential direction again, an oxidation peak A_2_ was detected at −0.15 V. This is associated with the oxidation of the produced H_2_S (at C_3_) back to elemental sulfur (S^0^), as expressed in reaction 8 (reverse of reaction 7). The intensity of peak A_2_ is anticipated to be low because the H_2_S_(g)_ generated is dispersed fairly quickly, resulting in its concentration being low near the electrode.[^18^, ^21^, ^34^]
(8)

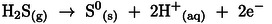




The oxidation peak A_3_ at +0.05 V, represents initial, partial oxidation of FeS_2_ to Fe(OH)_3_/Fe^3+^, Fe^2+^ and S^0^, based on reactions 9 and 10. Partial oxidation may also result in sulfur‐rich intermediate products, i. e. iron deficient sulfide (Fe_1‐x_S_2_) and polysulfides (S_n_^2−^). The accumulation of these substances on the electrode's surface leads to a diffusion layer, which only at higher potentials (at A_1_) can further oxidise to other species including SO_4_^2−^ and thus eliminate the diffusion barrier.[^18^, ^21^, ^31^, ^34^]
(9)

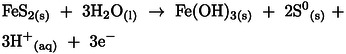



(10)

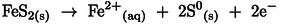




### 2.3 Cyclic Voltammetry (CV) for Chalcopyrite

**Figure 7:**
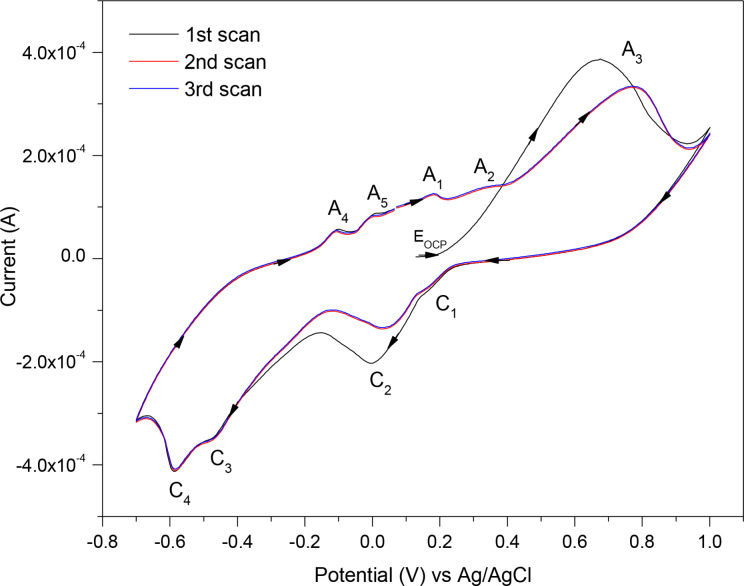
A three‐cycle CV scan of chalcopyrite mineral as the WE, measured at 0.02 V/s scan rate in 0.5 M HNO_3_.

A similar set of experiments was run on the freshly polished chalcopyrite electrode in 0.5 M HNO_3_ and the three‐cycle CV scan is presented in Figure 7. The scan was initiated from the E_OCP_=~+0.15 V (as marked in the graph) in the anodic direction to +1 V, then reversed in the cathodic direction to −0.7 V. In the initial anodic sweep from E_OCP_ only peak A_3_ was detected, but on the subsequent anodic scans peaks A_4_ and A_5_ as well as A_1_ and A_2_ were observed (five anodic peaks in total). On the subsequent scans peak A_3_ is also shifted in a more positive direction. Furthermore, in the cathodic part, four reduction peaks appeared.

**Figure 8:**
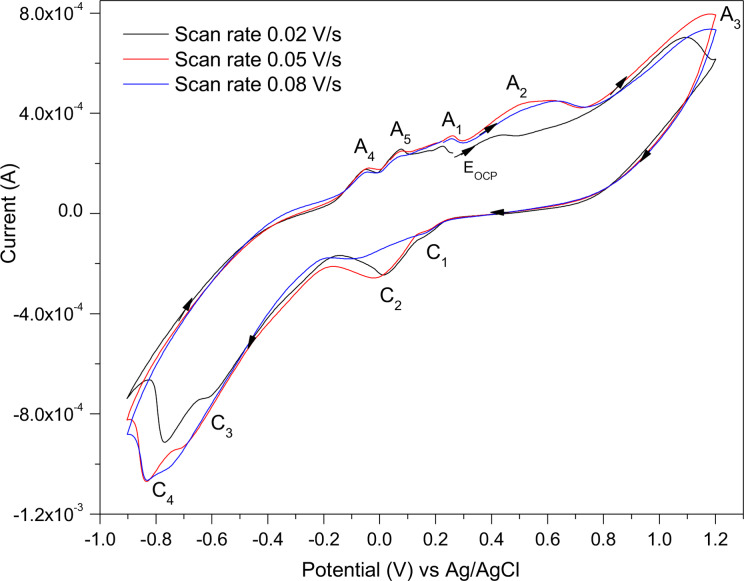
The CV scans for chalcopyrite as the WE at different scan rates in 0.5 M HNO_3_.

The voltammograms (for the third scan) of chalcopyrite at different scan rates of 0.02, 0.05, and 0.08 V/s are displayed in Figure 8. Where a faster scan rate resulted in more pronounced peaks for the pyrite electrode, the opposite was true for chalcopyrite with the slower scan rate of 0.02 V/s generally producing more pronounced redox peaks, thereby indicating which redox processes occur at slower rates. A scan rate of 0.02 V/s was applied in the subsequent CV scans.

#### 2.3.1 Effect of Changing Potential Range on Chalcopyrite

**Figure 9:**
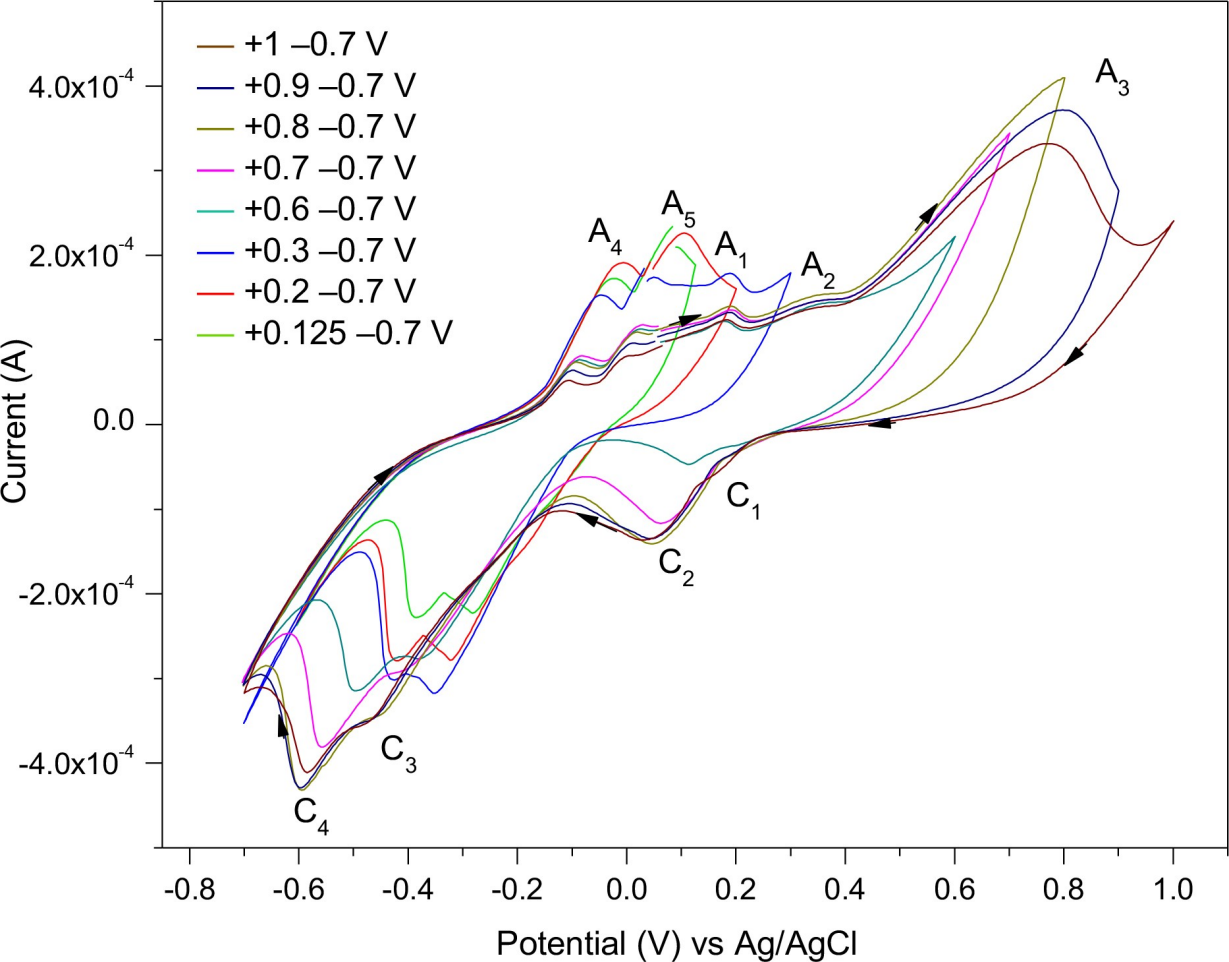
Effect of sweeping to different potentials in the positive range of the CV scans (the 3^rd^ cycles) of the chalcopyrite as the WE, at 0.02 V/s scan rate in 0.5 M HNO_3_.

The effect of reducing the vertex potential in the positive range to +1, 0.9, 0.8, 0.7, 0.6, 0.3, 0.2, and 0.125 and measuring to −0.7 V on the CV scans of chalcopyrite are illustrated in Figure 9. By reducing the positive potential range, the anodic reactions at A_3_ and A_2_ do not fully occur, hence this affects the cathodic reactions occurring at C_1_ and C_2_, as the current of these cathodic peaks reduced noticeably. Thus, by observing these CV scans with a gradual reduction in the positive potential range, one can deduce that the anodic reactions at A_3_ and A_2_ are related to the cathodic reactions at C_1_ and C_2_.

**Figure 10:**
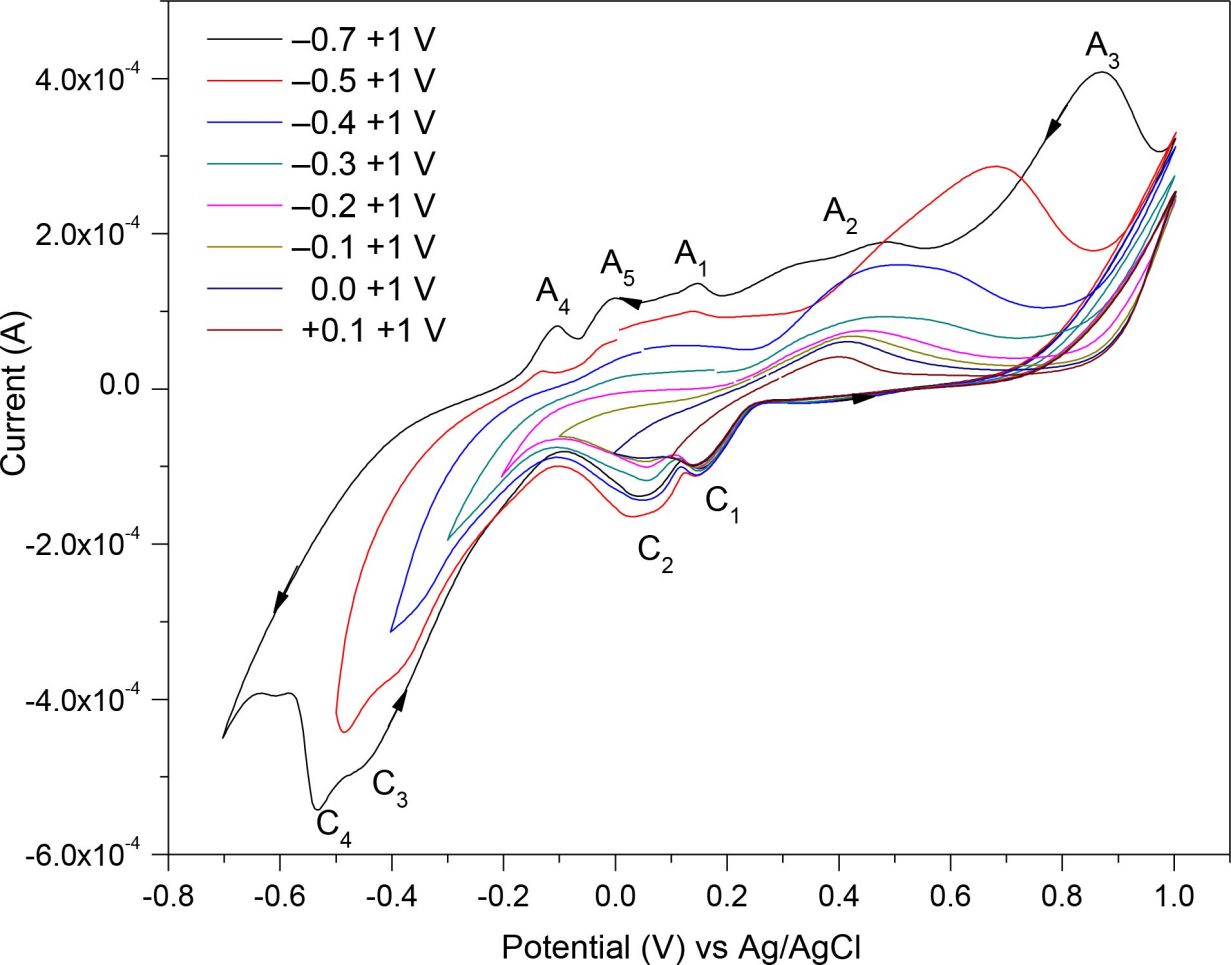
Effect of sweeping to different potentials on the negative range on the CV scans (the 3^rd^ cycles) of the chalcopyrite as the WE measures at 0.02 V/s scan rate in 0.5 M HNO_3_.

The effect of sweeping to different negative vertex potentials, commencing at E_ocp_ and sweeping to −0.7, −0.5, −0.4, −0.3, −0.2, −0.1, 0.0, and +0.1 V before reversing to a fixed positive potential at +1 V in the CV scans of the chalcopyrite electrode, are depicted in Figure 10. Reducing the negative potential range, the cathodic reactions at C_4_ and C_3_ do not occur fully, hence the anodic reactions at A_4_ and A_5_ are not driven to completion, thus the associated peaks (A_4_ and A_5_) appeared smaller. Further reducing the negative vertex potential affects the subsequent redox reactions occurring at A_1_, A_2_ and A_3_, as well as C_2_ and C_1_, as the detected redox peaks become smaller. Therefore, reducing the negative potential range gradually, indicated that the cathodic reactions at C_4_ and C_3_ are associated with anodic reactions at A_4_ and A_5_, and the cathodic reactions at C_2_ and C_1_, are related to reactions happening at A_1_, A_2_ and A_3_.

#### 2.3.2 Interpretation of the Redox Peaks in Chalcopyrite CV Scan

The current increased fairly rapidly during the anodic sweep soon after starting from E_OCP_=~+0.15 V, to give the peak A_3_ (see Figure 7). On subsequent scans an anodic peak A_1_ was detected at +0.2 V. This can be attributed to the partial oxidation of chalcopyrite (CuFeS_2_), where the preferential dissolution of Fe over Cu and S takes place to form copper‐sulfur‐rich species such as the nonstoichiometric segment Cu_1‐x_ Fe_1‐y_ S_2‐z_ (reaction 11), covellite (CuS) (reaction 12), and CuS_2_ (reaction 13). Although reaction 13 shows CuS_2_ as the product, the stoichiometry may not be exactly 1 : 2 as given here, but a more sulfur‐rich product, since some Cu can be oxidised as shown in reaction 11. The produced S^0^ (in reactions 11 and 12) can be related to a sulfur‐rich layer containing sulfur intermediate products, i. e. polysulfides (S_n_^2−^). Accumulation of these products leads to a thin passive layer partially covering the electrode's surface, which slows the dissolution rate down.[^17^, ^19^, ^20^, ^21^, ^35^]
(11)

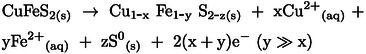



(12)

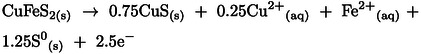



(13)

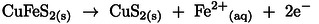




Peak A_2_ appeared like a broad shoulder at around +0.35 V. This is associated with the stepwise oxidation of Cu‐rich chalcocite (Cu_2_S) (produced in the reduction process C_1_) to a series of Cu‐deficient and non‐stoichiometric sulfides such as djurleite (Cu_1.92_S), digenite (Cu_1.6_S), and covellite (CuS) based on reactions 14, 15, and 16.[^17^, ^21^, ^36^]
(14)

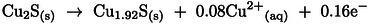



(15)

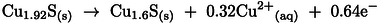



(16)

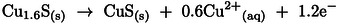




From ~0.6 V, a sharp escalation in anodic current was observed and the oxidation peak A_3_ appeared at 0.9 V (more positive than the initial peak for A_3_ seen on the very first scan), indicating oxidation of chalcopyrite (CuFeS_2_) to Cu^2+^, Fe^3+^ and S^0^/SO_4_^2−^, based on reactions 17 and 18, as well as further oxidation of CuS to Cu^2+^ and S^0^/SO_4_^2−^, according reactions 19 and 20.[^17^, ^21^, ^35^, ^37^]
(17)

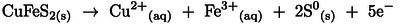



(18)

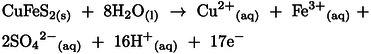



(19)

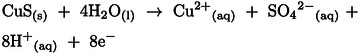



(20)

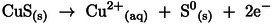




Reversal of the potential to the cathodic direction results in four discrete reduction peaks. The first reduction peak C_1_ identified at ~0.2 V, indicates the reduction of S^0^, thereby reincorporation of Cu^2+^ to form covellite (CuS) and further chalcocite (Cu_2_S) as written in reactions 21, 22 and 23, as well the reduction of Cu^2+^ to Cu^0^ (reaction 24.[^20^, ^21^, ^32^]
(21)

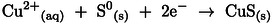



(22)

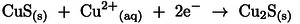



(23)

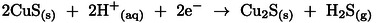



(24)

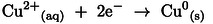




The second reduction peak C_2_ detected at ~0.05 V represents the reduction of remaining chalcopyrite at the surface according to three different possible reactions: (1) reduction to chalcocite (Cu_2_S), (2) reduction to bornite (Cu_5_FeS_4_), and (3) reduction to Cu_5_FeS_4_ and hydrogen sulfide (H_2_S), according to reactions 25, 26 and 27, respectively.[^17^, ^19^, ^21^] The reduction at C_1_ involves two electrons while that at C_2_ involves four electrons, hence the height of C_2_ is bigger than C_1_, as expected.[^17^, ^38^, ^39^]
(25)

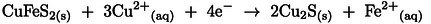



(26)

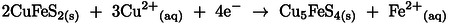



(27)

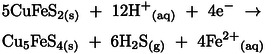




As the potential moved further in a negative direction, the cathodic current increased significantly after −0.1 V. The reduction peaks C_3_ and C_4_ appeared close to each other at around −0.45 V and −0.55 V, respectively. At peak C_3_ (−0.45 V) CuFeS_2_ and Cu_5_FeS_4_ can undergo further reduction to Cu_2_S, H_2_S, and Fe^2+^ as expressed in reactions 28 and 29.[^17^, ^21^, ^37^, ^38^, ^39^]
(28)

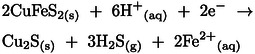



(29)

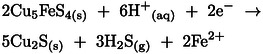




At peak C_4_ (at −0.55 V) CuFeS_2_ and Cu_2_S can undergo further reduction to form elemental copper (Cu^0^) and hydrogen sulfide (H_2_S), as articulated in reactions 30 and 31.[^17^, ^21^, ^37^]
(30)

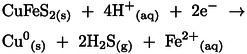



(31)

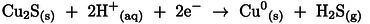




When the scan switched to the positive potential direction again, two oxidation peaks A_4_ and A_5_ were detected nearby at −0.15 V and −0.05 V, respectively. This is related to the oxidation of some of the reduction products formed during chalcopyrite reduction. Peak A_4_ is associated with the oxidation of Cu^0^ and H_2_S to chalcocite (Cu_2_S) according to reaction 32 (reverse of reaction 31). Peak A_5_ is related to the oxidation of H_2_S to elemental sulfur (S^0^) based on reaction 33.[^21^, ^38^, ^39^]
(32)

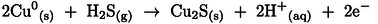



(33)

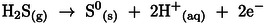




It should be mentioned that the slight differences in the position and height of the redox peaks in this study on pyrite and chalcopyrite electrodes compared to previous research could be due to small variations in the studied mineral chemical composition, possible impurities, and surface roughness, as well as the electrolyte used.^[18]^

### 2.4 Linear Sweep Voltammetry Study (LSV)

**Figure 11:**
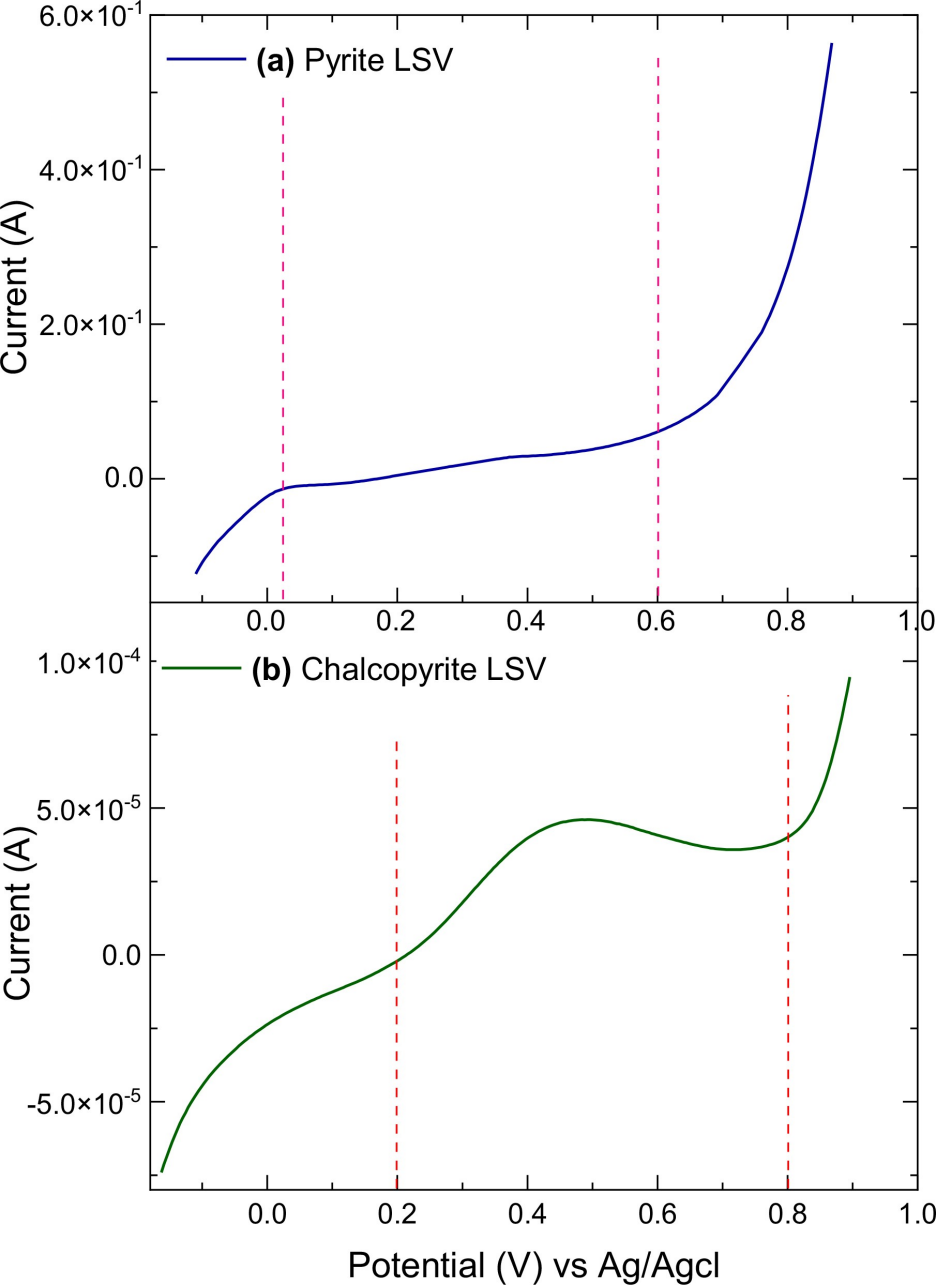
The linear sweep voltammetry (LSV) curves of (a) the pyrite electrode at 2 V/s scan rate and (b) the chalcopyrite electrode at 0.02 V/s scan rate in 0.5 M HNO_3_.

Figure 11. (a‐ pyrite and b‐ chalcopyrite) represents the linear sweep voltammetry (LSV) of a freshly polished pyrite electrode at 2 V/s scan rate and chalcopyrite electrode at a 0.02 V/s scan rate in 0.5 M nitric acid solution. The voltammogram for pyrite (11‐a) displayed three distinctive parts, similar to the research reported by Liu et al.^[31]^ and Tu et al..^[18]^ In the first region (−0.1–0.05 V), the current increases almost linearly with increasing potential. The voltammogram begins with a small negative current which could be associated with the reduction of pyrite (FeS_2_) to H_2_S (reaction 6). Then the oxidation of H_2_S to S^0^ (reaction 8 at A_2_) occurs initiating the anodic current. In the second region (about 0.1 V to 0.55 V), the current is somewhat independent of the increasing potential and increases only slowly. This could be related to the partial oxidation of pyrite (reaction 9 at A_3_) due to formation of Fe(OH)_3_, and a sulfur‐rich‐layer containing sulfur (S^0^), polysulfides (S_n_^2−^), and iron deficient sulfide (Fe_1‐x_S_2_), which, due to their accumulation on the electrode surface, form a thin passive layer that slows down oxidation, acting as rate‐limiting layer. In the third section (from 0.6 V to above 0.8 V), the current increased sharply with increasing potential, due to the improved reaction‐driven force. Thus, a potential higher than +0.6 V is sufficient to convert the insoluble species forming the inhibiting layer to soluble ions, thus eliminating the rate‐limiting surface layer. The high potential at this region can also lead to extensive pyrite oxidation which corresponds to the CV curve of pyrite at A_1_.[^18^, ^21^, ^30^, ^31^]

Figure 11‐b illustrates the LSV of the chalcopyrite electrode at a 0.02 V/s scan rate in 0.5 M nitric acid solution. Generally, three main sections were presented in the chalcopyrite LSV curve corresponding to the CV scan with the main anodic peaks. The first part which is at the lower anodic potential (−0.1 V to +0.2 V) displays a relatively passive section, where the current does not increase significantly with increasing potential. In this part, the voltammogram begins with a small negative current indicating the reduction of CuFeS_2_ to H_2_S and Cu^0^ (reaction 30). This section could be associated with the anodic reaction at A_5_, at which the oxidation of H_2_S to S^0^ (reaction 33) occurs and partially covers the surface of the electrode.[^17^, ^19^, ^21^, ^35^] In the second region (+0.2 V to +0.8 V), the current rises gently and is accompanied by two oxidation reactions, (1) the partial oxidation of chalcopyrite at A_1_, forming a non‐stoichiometric thin passive layer (Cu_1‐x_ Fe_1‐y_ S_2_) and, (2) a series of oxidation reactions of Cu_2_S to CuS and Cu^2+^ (at A_2_), which causes the moderate upsurge in the oxidative current. From the potential values of +0.6 V to +0.8 V, the current was constant, which can be due to the accumulation of these copper‐rich ‐sulfur species, forming another passive surface layer which reduces the oxidation rate.[^17^, ^19^, ^38^, ^39^] A subsequent sharp increase in the oxidative current happened after +0.8 V, proving the extensive oxidation of chalcopyrite (at A_3_) by releasing several electrons which results in a noticeable increase in the current in a short increment of increased potential.[^17^, ^37^]

### 2.5 Open Circuit Potential (OCP)

**Figure 12:**
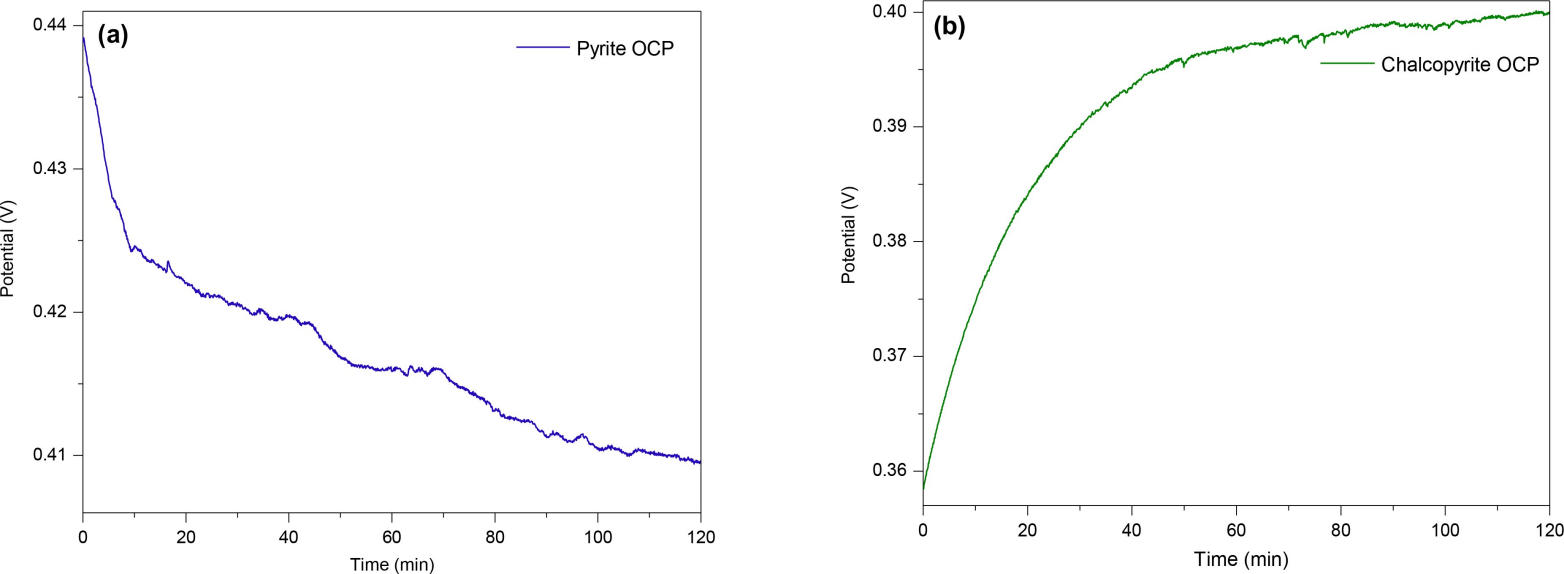
The open circuit potential (OCP) of (a) pyrite and (b) chalcopyrite as the working electrode in 0.5 M HNO_3_ for 120 min.

Before EIS measurements were conducted, the open circuit potential (OCP) was monitored to ensure the system had effectively reached thermodynamic equilibrium which took about 120 min. Figure 12 represents the OCP of freshly polished (a) pyrite and (b) chalcopyrite electrodes as a function of time. For pyrite, the OCP started from around 0.44 V and gradually reduced and stabilised at ~0.41 V, with an overall change of ~0.03 V (30 mV) in 120 min. The OCP for chalcopyrite began at ~0.36 V and increased before stabilising at ~0.40 V, with a total variation of ~0.04 V (40 mV).

### 2.6 Electrochemical Impedance Spectroscopy (EIS) Study for Pyrite and Chalcopyrite

Electrochemical impedance spectroscopy (EIS) is a powerful technique to provide supporting evidence for the product(s) formed as a result of an electrochemical reaction(s) at a specific potential on the surface of the working electrode.^[19]^ In the case of pyrite and chalcopyrite as the working electrodes, several oxidation peaks were detected in the anodic part of their CV scans. Therefore, to examine these processes more closely, EIS measurements were conducted using specific DC bias potentials with a 10 mV AC amplitude.

**Figure 13:**
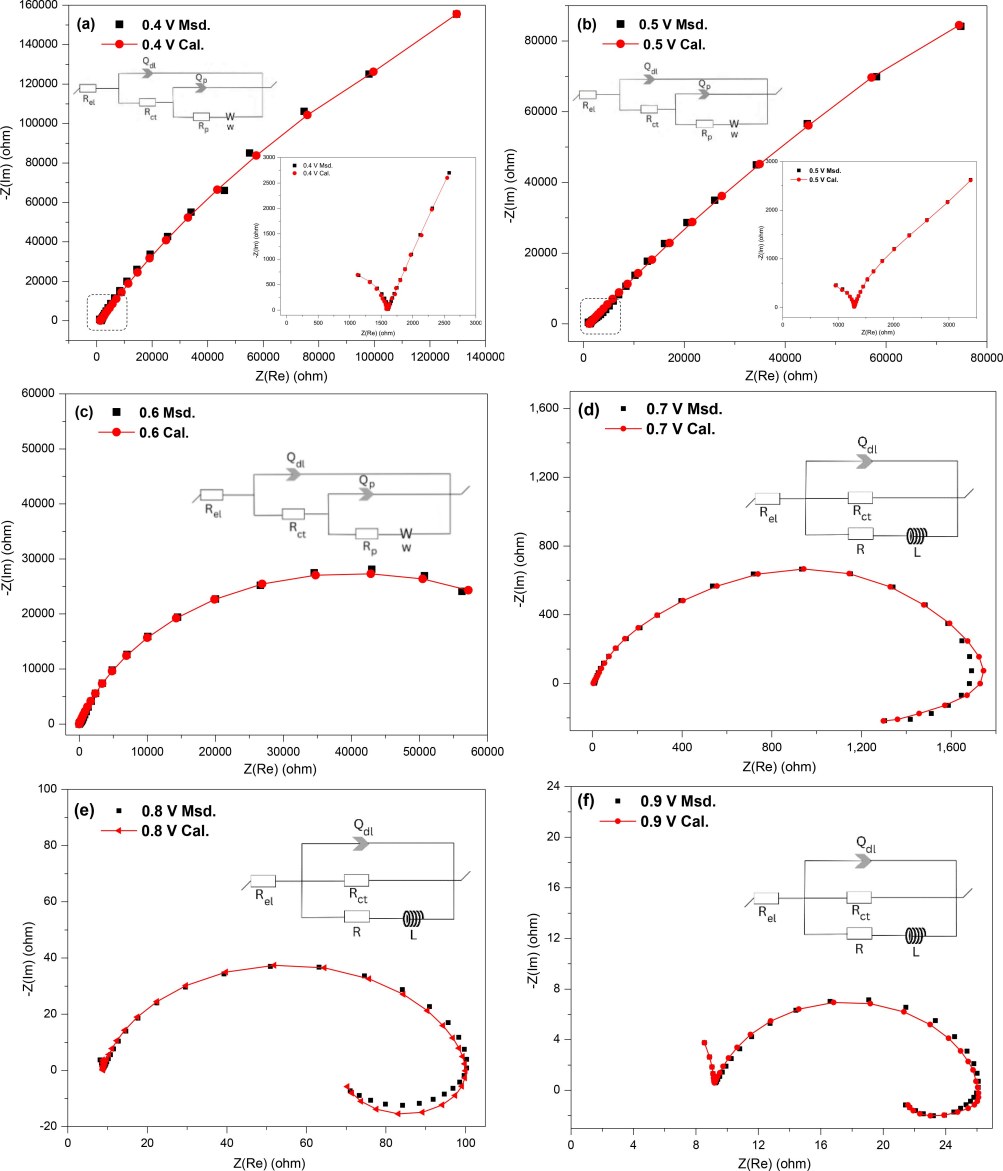
The measured and calculated Nyquist plots with the corresponding circuit models for pyrite in 0.5 M HNO_3_ at different bias potentials (a: 0.4 V (OCP), b: 0.5 V, c: 0.6 V, d: 0.7 V, e: 0.8 V, f: 0.9 V).

Figure 13(a–f) presents the Nyquist plots and fitted data using the indicated equivalent circuits for pyrite at 0.4, 0.5, 0.6, 0.7, 0.8, and 0.9 V. The Nyquist plots for the pyrite electrode at low anodic potentials (0.4 and 0.5 V) displayed a slight shift along the x‐axis, followed by a very small incomplete semicircle in the high‐frequency region, and a largely straight line indicating a typical Warburg impedance (W) feature in the middle and low‐frequency region. The shift along the x‐axis represents the resistance of the electrolyte solution (R_el_), and the small incomplete semicircle in the high‐frequency region represents the charge transfer resistance (R_ct_) and interfacial double layer capacitance (Q_dl_) represented by a constant phase element due to inhomogeneities across the surface. The Warburg impedance (W) corresponds to the presence of a diffusion process (semi‐infinite diffusion).[^18^, ^30^, ^31^] This can be due to the produced Fe(OH)_3_ and S^0^ (or other sulfur‐rich‐layers) during partial oxidisation of pyrite (as at A_3_) covering the surface of the pyrite electrode, making the process diffusion controlled at these low frequencies.^[18]^ The equivalent circuits used to fit the data are given in Figure 13(a and b) were [R_el_+Q_dl_/(R_ct_+Q_p_/(R_p_+W)], with R_p_ the passive layer resistance and Q_p_ the capacitance associated with that passive layer (also represented by a constant phase element). This is due to the products formed during the partial oxidation of pyrite partially covering the electrode's surface.[^18^, ^30^, ^31^]

The Nyquist plot at 0.6 V (Figure 13‐c) shows a trivial shift along the x‐axis presenting the resistance of the electrolyte solution (R_el_). This is followed by a small incomplete semicircle in the high‐frequency region associated with Q_dl_ and R_ct_. The middle to the low‐frequency region of the depressed curve indicates the Warburg element (W), which represents more bounded diffusion signifying a thinner passive layer as it is oxidised when moving into the transpassive region.[^18^, ^31^]

The Nyquist plots at 0.7, 0.8, and 0.9 V (Figure 13d–f) were similar to each other, except for the magnitude of the impedance which decreased with increasing bias potential. At high frequencies, the Nyquist plots again show a small shift along the x‐axis indicative of R_el_, and a small incomplete arc indicative of Q_dl_ and R_ct_. In the low‐frequency region, the curve extended to the fourth quadrant, indicating a clear inductive feature (L). Tu et al.^[18]^ and Lui et al.^[31]^ associated this with the adsorption and desorption of some sulfide species produced during the rapid and extensive anodic dissolution of pyrite at high potentials (A_1_). However, since induction involves the ability to store magnetic energy, another possibility could be the existence of species whose transient magnetisation energy is being observed at high frequencies. No specific diffusion process was evident, again indicating that the oxidative driving force above 0.6 V is adequate to remove the accumulated species from the electrode's surface. At higher potentials, the circuit including the inductive reactant (L) and R, which is the resistance associated with the inductance process as [R_el_+Q_dl_/R_ct_ (R+L)], was used for the equivalent circuit.

Table 1 summarises the results for the parameters in the equivalent circuits at the studied potentials for pyrite. As expected the values for R_el_ (the resistance of the 0.5 M HNO_3_ solution) were all similar at ~8 Ω. The charge transfers resistance (R_ct_), associated with the rate of electron transfer for the different anodic reactions, showed a decreasing trend when increasing the bias potentials, implying a faster rate of pyrite oxidation. Similarly, the values for the resistance related to the surface passive layer (R_p_) decreased by increasing the potentials. The high R_p_ values at lower potentials (especially 0.4 and 0.5 V) signify the high resistance due to the passive layer partially covering the surface, while increasing the potential led to anodic reactions which eliminated the passive layer. Furthermore, the values for n define the degree of deviation for the double‐layer capacitance (Q_dl_, presented by a constant phase element) from the ideal capacitance (C, at n=1). By increasing the applied potential, the values for n gradually declined. This indicates that at higher potentials, the surface of the electrode becomes rougher due to the rapid dissolution reactions, and also more inhomogeneous and thus more distributed reaction rates occur. Hence, the higher the potential, the smaller the value of n. Moreover, the small values for $\chi^{2}$ /lZl indicate a decent fit between the experimental and fitted values using the stated equivalent circuits.[^18^, ^31^, ^34^]

**Table 1:** Values of the equivalent circuit elements at the studied bias potentials for pyrite.

Applied potential (V)^]^	R_el_ (Ω)	Q_dl_ (10^−6^ F s^n^)	n	R_ct_ (Ω)	Q_p_ (10^−6^ F s^n^)	n	R_p_ (Ω)	W (10^−6^ Ω s^−0.5^)	L (H)	$\chi^{2}$ /lZl
0.4	8.9	1.2	0.81	1.87×10^3^	25.7	0.79	5.64×10^5^	3.11×10^4^	–	0.22
0.5	8.7	1.7	0.72	1.30×10^3^	21.6	0.68	4.92×10^5^	1.64×10^4^	–	0.43
0.6	8.5	5.3	0.76	1.04×10^3^	11.2	0.72	7.05×10^4^	1.56×10^3^	–	0.37
0.7	8.4	0.56	0.71	9.09×10^2^	–	–	*3.76×10^3^	–	28.6	0.42
0.8	8.3	0.43	0.66	92.3	–	–	*174.9	–	17.4	0.36
0.9	8.2	0.41	0.62	17.1	–	–	*44.2	–	6.7	0.32

R_el_ is the electrolyte resistance, Q_dl_ is the capacitance for the double layer, R_ct_ is the charge transfer resistance, Q_p_ is the capacitance for the passive layer, n is the degree of deviation from ideal capacitance (C, n=1), R_p_ is the resistance of the passive surface layer, W is Warburg impedance, L is an inductive element, and *is R_L_ the resistance related to the inductive process.

**Figure 14:**
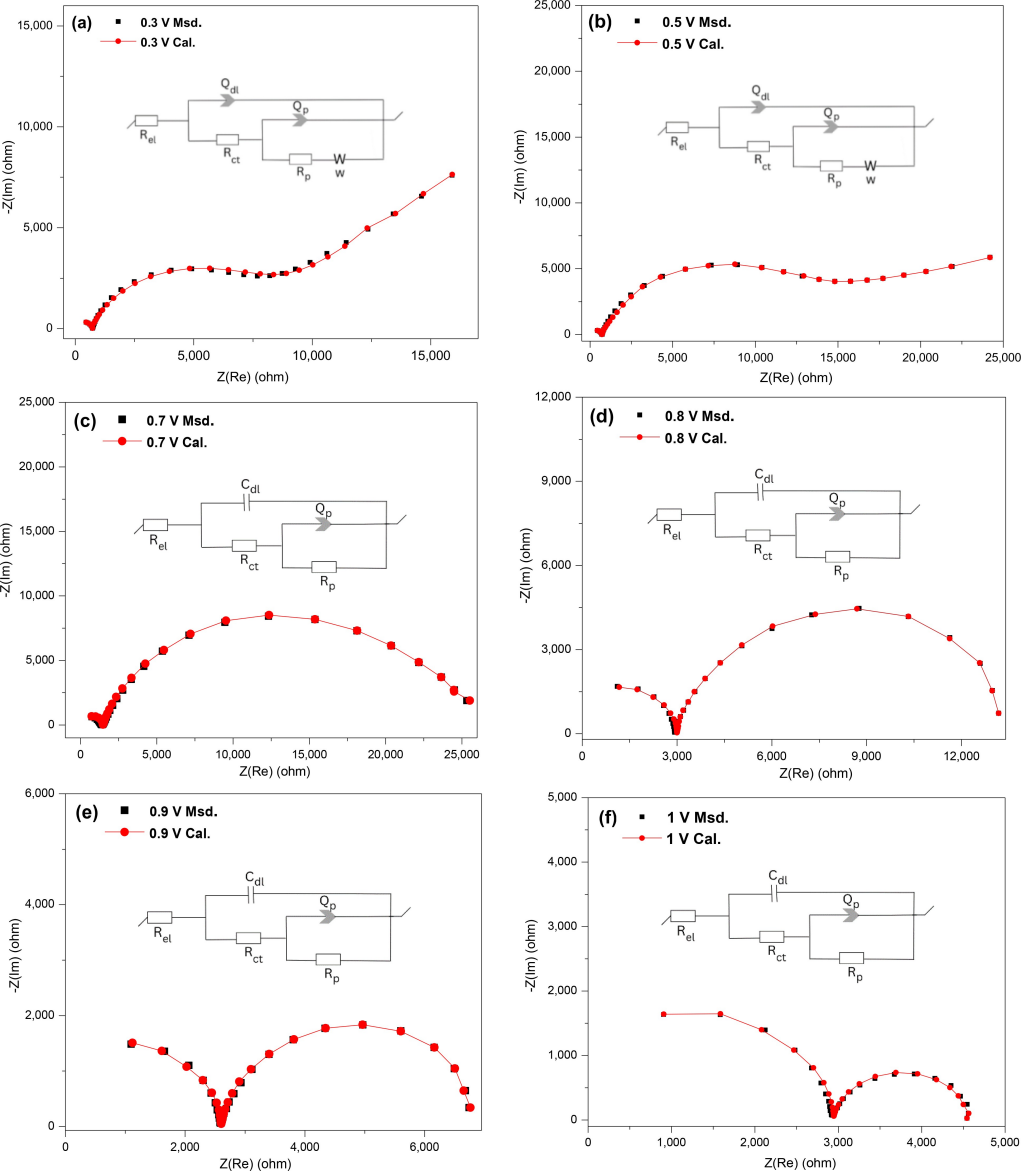
The experimental measured and calculated Nyquist plots with the corresponding circuit models for chalcopyrite in 0.5 M HNO_3_ at different potentials (a: 0.3 V, b: 0.5 V, c: 0.7 V, d: 0.8 V, e: 0.9 V, f: 1 V).

Figure 14(a–f) presents the Nyquist plots and fitted data using the indicated equivalent circuits for chalcopyrite at 0.3–1 V. All the plots start with a slight shift along the x‐axis indicative of R_el_ and a small incomplete semi‐circle in the high‐frequency region related to the double layer capacitance (C_dl_ or Q_dl_) and the resistance for the main charge transfer (R_ct_). At low bias potentials (0.3 and 0.5 V), there is a semicircle in the mid‐frequency range correlated with the capacitance (which shows a distributed behaviour due to an inhomogeneous surface) and resistance of the surface passive layer (denoted Q_p_ and R_p_, respectively), which is followed by a straight line indicating Warburg diffusion (W) in the low‐frequency region. The species covering the surface of the chalcopyrite electrode at these potentials, which account for the diffusion process, likely are predominantly iron‐deficient sulfide leaving a copper‐rich‐sulfur passive layer (i. e. Cu_1‐x_ Fe_1‐y_ S_2‐z_, Cu_1‐z_S_2_, CuS_2_ and CuS) and S^0^.[^17^, ^19^, ^20^] As the bias potential was increased from 0.3 V to 0.5 V, the resistance of the passive layer (R_p_) decreased, thus indicating less resistive oxidative reactions at 0.5 V. Also the angle of the straight line in the high‐frequency region (Warburg diffusion) is deviating from 45 degrees, which could be related to the diffusion process at 0.5 V at which the semi‐infinite condition is slightly changing. The equivalent circuits used to fit the data given in Figure 14(a and b) were [R_el_+Q_sl_/(R_ct_+Q_dl_/(R_p_+W)].

At high anodic potentials (0.7–1 V), the Nyquist plots start with an incomplete semi‐circle in the high‐frequency region as before, followed by a semicircle in the middle to low‐frequency regions with no evidence of the semi‐infinite Warburg diffusion. The resistance associated with the middle to low‐frequency semicircle decreased as the bias potential was increased, indicating the oxidative dissolution of the copper‐rich‐sulfur layer and S^0^ to form soluble species.[^17^, ^19^, ^20^]

Table 2 gives the values of all of the elements from the equivalent circuits at the studied potentials for chalcopyrite. As was anticipated, the values for the electrolyte solution resistance (R_el_) were close to one another because the same solution was used for the different potentials. Increasing the anodic potentials from 0.2–1 V, showed a decreasing trend for the resistance related to the surface passive layer (R_p_). This suggests that the total impedance decreased and subsequently the oxidation rate increased. Raising the applied potentials can cause the charge transfer resistance (R_ct_) to fluctuate, which could be due to the different complicated electrochemical reactions occurring on the surface of chalcopyrite electrode at each specific potential.[^17^, ^19^, ^20^]

**Table 2:** Values of the elements from the equivalent circuits for the studied potentials for chalcopyrite.

Applied potential (V)^]^	R_el_ (Ω)	Q_dl_/C_dl_ (10^−7^ F s^n^)	n	R_ct_ (Ω)	Q_p_ (10^−6^ F s^n^)	n	R_p_ (Ω)	W(Ω s^−0.5^)	$\chi^{2}$ /lZl
0.3	7.4	2.5	0.81	7.27×10^2^	14.5	0.78	1.62×10^4^	1.99×10^3^	0.21
0.5	7.3	2.4	0.86	6.35×10^2^	18.4	0.76	1.57×10^4^	1.51×10^3^	0.32
0.7	7.7	0.34	1	1.25×10^3^	7.7	0.84	1.41×10^4^	–	0.36
0.8	7.6	0.83	1	2.99×10^3^	3.7	0.86	1.04×10^4^	–	0.39
0.9	7.4	1.6	1	2.52×10^3^	2.4	0.83	4.25×10^3^	–	0.23
1	7.1	1.4	1	2.87×10^3^	1.1	0.81	1.64×10^3^	–	0.28

R_el_ is the electrolyte resistance, Q_dl_/C_dl_ is the capacitance for the double layer, R_ct_ is the charge transfer resistance, Q_p_ is the capacitance for the passive layer, n is the degree of deviation from ideal capacitance (C, n=1), R_p_ is the resistance of the passive surface layer, and W is Warburg impedance.

In summary, the results determined from the EIS data for pyrite and chalcopyrite at different potentials correspond to the reactions taking place during anodic dissolution as detected in their CV scans at the specific potentials. For both pyrite and chalcopyrite electrodes the anodic dissolution at low potentials (0.4–0.6 V for pyrite and 0.3–0.5 V for chalcopyrite) starts with preferentially dissolving iron, hence forming sulfur‐rich species (Fe_1‐x_S_2_, S^0^)^[18]^ and copper‐rich sulfur species (Cu_1‐x_Fe_1‐y_S_2‐z_, Cu_1‐z_S_2_, CuS_2_, CuS), respectively.[^17^, ^19^, ^23^, ^36^, ^37^] These nonstoichiometric species accumulate and partially cover the electrode's surface acting as a passive layer, resulting in a diffusion barrier, and slowing down the oxidation process. The passive layer was confirmed with the existence of a Warburg diffusion (W) element, as indicated in the equivalent circuits at low‐potentials for both pyrite and chalcopyrite.

When the bias potential is increased, the charge transfer resistance at higher frequencies for pyrite decreases, contrary to that observed for chalcopyrite, indicating that the passive layer is oxidised more readily for pyrite in 0.5 M HNO_3_. Increasing the potential to above 0.7 V the species in the passive layer further oxidise, thereafter eradicating the diffusion layer for both minerals, and leading to faster oxidation, more so for pyrite. This is supported by the observations that, when measuring the CV scans for chalcopyrite, the scan rates had to be slow (~0.02 V/s) for the range of redox processes to be seen indicating kinetically slow processes. On the other hand, fast scan rates (~2 V/s) were used to measure the CV scans for pyrite. Additionally, when the potential on the CV scans was increased above 0.7 V, the current increases exponentially for pyrite but a peak maximum is reached for chalcopyrite.

At higher potentials, different equivalent circuits were needed to fit the Nyquist plots for pyrite and chalcopyrite. For pyrite, a low‐frequency inductive feature (L) was present for measurements at high potentials (0.7 V to 0.9 V), previously attributed to adsorption and desorption of sulfide species produced during the rapid and extensive anodic dissolution of pyrite,^[18]^ but which could also be due to transient magnetised species. This inductive feature was not seen in the Nyquist plots for chalcopyrite, which instead had a second semi‐circle at low frequency, where the magnitude of the resistance decreased as the potential was raised to 1 V, signifying increased dissolution. Hence different mechanisms for dissolution were apparent for pyrite and chalcopyrite at high potentials.

These results were compared to similar studies performed in H_2_SO_4_ solutions, a commonly used acid for the dissolution of pyrite and chalcopyrite. The following observations are noted: (i) Both HNO_3_ and H_2_SO_4_ are acids capable of dissolving these minerals, with HNO_3_ being a stronger oxidant due to N being more electronegative than S. Thus the oxidative dissolution of these minerals in both these acids can break down the host mineral to expose any encapsulated gold and improve its recovery. (ii) Pyrite dissolves faster in HNO_3_ than H_2_SO_4_, however, the latter is more suitable for chalcopyrite dissolution. This is due to the passive layer formed on chalcopyrite dissolving more readily in H_2_SO_4_ than HNO_3_, although higher potentials are required in H_2_SO_4_ (as compared to that in HNO_3_)[^23^, ^40^] (iii) The dissolution kinetics for both pyrite and chalcopyrite is diffusion‐controlled in HNO_3_ and H_2_SO_4_. (iv) Pyrite oxidation occurs faster (with a higher anodic and cathodic current density being noted) compared to chalcopyrite in HNO_3_. The slower chalcopyrite dissolution is likely caused by the formation of copper‐rich‐sulfur species (such as Cu_1‐x_Fe_1‐y_S_2‐z_, CuS_2_ and Cu_1‐z_S_2_), which also slows the diffusion of Fe ions through these formed passive layers.[^27^, ^40^]

## 3 Conclusions

Understanding the oxidation of pyrite and chalcopyrite is an important issue because of exposing any encapsulated gold to enhance its extraction yield from refractory ores and mine tailings. CV scans along with EIS measurements were employed to study the electrochemical behaviour of pyrite and chalcopyrite in 0.5 M nitric acid as the electrolyte. Anodic oxidation of pyrite (FeS_2_) at low potentials (0.4–0.6 V) begins with the oxidation of iron which results in the formation of Fe_1‐x_ S_2_, Fe(OH)_3_ and elemental sulfur (S^0^) that partially cover the surface of the pyrite electrode and cause a diffusion barrier, thus diminishing further anodic reactions. Increasing the potentials to above 0.7 V leads to reactions in which the products accumulated on the electrode's surface convert to soluble species, i. e. S^0^ to H_2_S or SO_4_^2−^, hence eradicating the diffusion barrier and leading to extensive oxidation of pyrite at high potentials.

The electrochemical oxidation of chalcopyrite was somewhat similar to that of pyrite since at low potentials (below 0.5 V), iron is preferentially dissolved (Fe), hence some copper rich‐sulfur species like Cu_1‐x_ Fe_1‐y_ S_2‐z_, CuS_2_, CuS and S^0^ formed and accumulated on the chalcopyrite electrode's surface to cause a passive layer. Increasing the potential to above 0.7 V leads to reactions at which the species in the formed passive layer are oxidised to soluble species, thereby eliminating the diffusion barrier. The Nyquist plots from the EIS measurements provide evidence supporting the reactions and the suggested products at the studied potentials. As a result, the existence of a semi‐infinite diffusion described by the Warburg element in fitting the EIS data at low potentials for both pyrite and chalcopyrite indicates a diffusion‐controlled dissolution process. Moreover, at high potentials (above 0.6 V) faster and more active dissolution of the mineral occurs, confirming the elimination of the passive layer covering the electrodes’ surface.

The oxidative dissolution of the pyrite in 0.5 M HNO_3_ occurs at a faster rate than that for chalcopyrite, with different dissolution processes occurring for the two minerals as evidenced by the differences in the EIS data when increasing the bias potentials. The copper‐rich sulfur layers and sulfur‐rich layers clearly influence the dissolution processes significantly.

## Funding

This research was funded by DRDGOLD (Pty.) Ltd. in South Africa.

## Conflict of Interests

The Authors declare that there is no conflict of interest in this work.

4

## Data Availability

The data that support the findings of this study are available from the corresponding author upon reasonable request.
